# Operating Within the Neonatal Intensive Care Unit: A Retrospective Analysis From a Tertiary Care Center

**DOI:** 10.7759/cureus.16077

**Published:** 2021-06-30

**Authors:** Sachit Anand, Gursev Sandlas, Neha Nabar, Preetha Joshi, Mohan Terdal, Shaila Suratkal

**Affiliations:** 1 Pediatric Surgery, Kokilaben Dhirubhai Ambani Hospital and Medical Research Institute, Mumbai, IND; 2 Neonatology, Kokilaben Dhirubhai Ambani Hospital and Medical Research Institute, Mumbai, IND; 3 Anaesthesiology, Kokilaben Dhirubhai Ambani Hospital and Medical Research Institute, Mumbai, IND

**Keywords:** bedside surgery, preterm newborn, very low birth weight, neonatal surgery, neonatal intensive care unit (nicu)

## Abstract

Background

Despite ongoing advances in the field of neonatology, the survival outcomes among critically ill preterm surgical neonates remain unfavorable. Intrahospital transport is one of the major risk factors associated with early mortality (within 30 days) in these newborns. To overcome this, the approach of performing bedside surgeries is being followed. We aim to assess the safety and feasibility of performing bedside neonatal surgeries by analyzing our archives.

Methods

The study focused on retrospective evaluation of all the newborns who have undergone surgical procedures in the neonatal intensive care unit (NICU) at our center from August 2015 through February 2021. Newborns were operated within the NICU if they had very low birth weight or other risk factors making their transport to the operation room risky. The outcomes of surgeries were assessed in terms of postoperative complications, one-month survival, and overall survival.

Results

Thirteen children (M:F=9:4) underwent twenty-two surgical procedures. The median (range) gestational age and birth weight of our cohort were 30 (26-36) weeks and 1200 (500-2860) grams, respectively. One-month and overall survival rates in our cohort were 84% (11/13) and 77% (10/13), respectively. No major postoperative complications were observed. The requirement of multiple inotropes and/or high-frequency oscillatory ventilation (HFOV) was the only factor having a significant association with unfavorable survival outcomes.

Conclusions

Bedside surgery is a safe and feasible alternative to surgeries within the operation room for at-risk newborns. In the present study, the requirement of multiple inotropes and/or HFOV was the only factor significantly associated with early mortality.

## Introduction

The care of a surgical newborn is extremely challenging and requires the continuous involvement of skilled neonatologists for favorable outcomes. Despite a better understanding of postnatal and transitional neonatal care, the mortality rates among surgical newborns can be as high as >60% in developing countries [1]. Numerous preoperative, intraoperative, and postoperative factors have been linked with poor survival outcomes in these newborns [1,2] One major risk factor is the transport of critically ill and/or preterm surgical newborns to and from the operation room (OR). It has been demonstrated that various unfavorable incidents including hypothermia, displacement of the endotracheal tube, dislodgements of the intravenous lines, etc. can happen during the intra-hospital transfer of these babies [2,3]. Also, newborns on high-frequency oscillatory ventilation (HFOV) and multiple inotropes in the preoperative period require close observation and their transport to OR is very risky. To overcome these, few neonatal intensive care units (NICUs) across the globe have adopted the approach of bedside surgery or surgery within the NICU [2-4]. Apart from minor procedures like insertion of intercostal drains, peritoneal dialysis catheters, and establishment of central venous access; major surgeries for patent ductus arteriosus (PDA), perforation peritonitis, intestinal obstruction, strangulated inguinal hernia, gastroschisis, congenital diaphragmatic hernia (CDH), etc. have been performed within the NICU with fair degrees of success [2,4,5]. A similar approach has been followed at our center where surgeries within the NICU are performed in a selected subset of newborns to ensure continuity of care by the same intensive care team. By reviewing the survival outcomes and postoperative complications, we aim to assess the safety and feasibility of performing surgeries within the NICU at our center. We also intend to determine the factors associated with unfavorable outcomes in newborns undergoing bedside surgeries.

## Materials and methods

Patient population

The study included all neonates who have undergone procedures for pediatric surgical conditions in the NICU at our center from August 2015 through February 2021. The majority of these procedures were performed by a senior Pediatric Surgeon. An ethical waiver was given by the Institute’s ethical committee in view of the retrospective nature of the study and all procedures being performed as a part of the routine management of these newborns. Data including antenatal diagnosis of the anomaly, gestational age, place of birth (outborn or inborn), birth weight, gender, age at the time of first surgery, number of surgeries per newborn, details of each surgical procedure, outcomes of surgery, and follow-up details were retrieved from the archives. The outcomes of surgeries were assessed in terms of postoperative complications, one-month survival, and overall survival.

Criteria of surgery within the NICU 

As per our institutional criteria, newborns are operated within the NICU when they have very low birth weight (VLBW), i.e. <1500 grams; or any birth weight with risk factors making their transport to the OR risky such as the requirement of multiple inotropes, need for high-frequency ventilation, etc. 

Preparation and planning of the surgical procedure 

There are a total of 15 beds including four isolation rooms in the NICU at our center. These isolation rooms have a room area of 120 square feet and laminar airflow with 12 air changes per hour. The newborn, who is planned for the surgical procedure, is either nursed in the isolation room only or is shifted from the adjacent NICU bed after negative swab cultures of the isolation room. The energy sources, instrument set, anesthetic drugs, and consumables are transferred to the NICU. The operating team consists of two surgeons, an anesthetist, a neonatologist, a scrubbed nurse, and an OR technician. The ventilator settings are adjusted as per the baby’s requirements and intravenous anesthesia is given throughout the procedure. Continuous monitoring of the vital parameters is ensured during the surgical procedure. After the surgery, the baby is kept in the isolation room until declared physiologically stable by the neonatologist.

Statistical analysis

Data were expressed as numbers, proportion, median, and range. The qualitative variables were analyzed using Fischer’s exact test. Factors significantly associated with early mortality (within one month of surgery) were assessed. A p-value of <0.05 was considered to be statistically significant. Data entry was done using Microsoft Excel (Microsoft Corporation, Redmond, USA) and analysis was performed using Stata/SE 12.0 (StataCorp, College Station, USA).

## Results

A total of thirteen newborns (four females) were included in the present study. Only five (38%) of them had an antenatal diagnosis of their surgical anomaly. The median (range) gestational age at the time of delivery was 30 (26-36) weeks with an outborn:inborn ratio of 9:4. The median (range) birth weight of our cohort was 1200 (500-2860) grams. Associated congenital anomalies were present in 54% (7/13) of the newborns. Ten babies (77%) required mechanical ventilation in the preoperative period. Table 1 depicts the patient characteristics.

**Table 1:** Patient characteristics

Patient characteristics	
Antenatally diagnosed; n (%)	5 (38%)
Median (range) gestational age; in weeks	30 (26-36)
Inborn delivery; n (%)	4 (31%)
Median (range) birth weight; in grams	1200 (500-2860)
Gender (M:F)	9:4
Median (range) age at first surgery; in days	3 (2-60)
Associated anomalies, n (%)	
Cardiac disease	7 (54%)
Unilateral cleft lip	1 (8%)

The surgeries were performed for perforation peritonitis (7/13; 54%), intestinal obstruction (2/13; 15%), feeding intolerance (2/13; 15%), and urinary tract abnormalities causing complicated urinary tract infections (2/13; 15%). Overall, 22 surgeries were done in the cohort at a median (range) age of 3 (2-60) days. Of these, 18 procedures (82%) were performed by the pediatric surgery team. The remaining four procedures (18%) including clipping of the patent ductus arteriosus (PDA) (n=3) and laser excision of the aryepiglottic fold (n=1) required the involvement of Cardiac and Otolaryngology surgery teams respectively. Table 2 highlights the diagnosis and surgical procedures performed in the present cohort.

**Table 2:** Details of diagnosis and surgical procedures in the cohort ^†^ and ^‡^ had severe gastroesophageal reflux disease with Tetralogy of Fallot and laryngotracheomalacia with large aortopulmonary window respectively. ^§^Both the newborns had urosepsis at the time of presentation. One of them had upper ureteral stricture while the other had ureterocele. ^¶^One of them underwent diagnostic laparoscopy followed by ileostomy creation due to distal ileal atresia.

Diagnosis and procedure	n (%)
Diagnosis	
Perforation peritonitis	7 (54%)
Intestinal obstruction	2 (15%)
Feeding intolerance^†‡^	2 (15%)
Complicated urinary tract anomalies^§^	2 (15%)
Surgical procedure	
Ileostomy^¶^	6/22 (27%)
Peritoneal drain insertion	5/22 (23%)
Feeding gastrostomy	2/22 (9%)
Repair of jejunal perforation	1/22 (4.5%)
Ileostomy closure	1/22 (4.5%)
Left pyelostomy	1/22 (4.5%)
Cystoscopy and deroofing of the ureterocele	1/22 (4.5%)
Bilateral inguinal herniotomy	1/22 (4.5%)

No major postoperative complications were observed in these newborns. Minor complications included wound dehiscence (3/13; 23%), persistent ileus (3/13; 23%), and peristomal excoriation (2/13; 15%). The one-month survival in our cohort was 84% (11/13). There were two early postoperative deaths due to refractory septic shock. Both of them had perforation peritonitis at the time of presentation. The only factor that was significantly associated (p=0.01) with early postoperative mortality was sick newborns on multiple inotropes and/or HFOV (Table 3). Other factors including gestational age <30 weeks, birth weight <1500 grams, specific gender, place of delivery (outborn/inborn), and associated cardiac anomalies had no significant association with poor survival outcomes. The overall survival of the study cohort after a median (range) follow-up of 46 (6-63) months was 77% (10/13). One infant, who was successfully discharged at four months of age, developed persistent gastroesophageal reflux. While on conservative management, he developed aspiration pneumonitis and died at the age of six months. 

**Table 3:** Factors associated with early postoperative mortality *Significant difference (p=0.01) HFOV: high-frequency oscillatory ventilation

Variable	Mortality (n=2)	No mortality (n=11)
Gestational age		
< 30 weeks	2	3
30 weeks or more	0	8
Gender		
Female	0	4
Male	2	7
Birth weight		
<1500 grams	2	7
1500 grams or more	0	4
Outborn/Inborn		
Outborn	1	8
Inborn	1	3
Associated cardiac anomaly		
Yes	2	5
No	0	6
Multiple inotropes/HFOV		
Yes	2	0
No	0	11*

## Discussion

Management of neonatal surgical conditions is challenging. For achieving the best outcomes, optimization of perioperative stress factors is required apart from a high degree of surgical precision. Factors significantly associated with poor survival outcomes after newborn surgery include preoperative - low birth weight, lesser gestational age, positive blood cultures, and associated congenital anomalies; intraoperative - prolonged duration of surgery, blood loss of >10% blood volume, and intraoperative hypothermia; and postoperative - a requirement of mechanical ventilation for a prolonged duration, need for high-dose of vasopressors, postoperative sepsis, major complications in the postoperative period, and prolonged fasting [1]. A number of these risk factors are linked to unnecessary intra-hospital or inter-hospital transport of newborns [2,6].

In the study by Beckmann et al., a total of 191 incidents were reported due to intra-hospital transport of critically ill patients. Of these, more than one-third were contributed by transport to or from the OR [7]. This becomes exceedingly critical among the surgical newborns where transport between the NICU and OR can lead to hypothermia, hyperglycemia, accidental displacements of the endotracheal tube, dislodgements of the intravenous lines, etc. [2]. These incidents can be reduced by performing surgeries within the NICU for a selected group of newborns. First introduced by Besag et al. [8] in two critically ill extremely low-birth-weight neonates, bedside surgery has gained immense popularity since then. At present, this approach has been incorporated into the protocols of few NICUs across the globe [2-5]. In fact, as per the recent survey of the Italian Society of Pediatric Surgery, bedside NICU surgery was reported by more than 80% of the respondent NICUs [4].

Critically ill preterm newborns are considered to be the prime candidates for bedside surgery as their transport to OR is very risky. However, the selection criteria in terms of birth weight differ among different NICUs. While some centers follow a cutoff of 1000 grams, others believe newborns with birth weight <1200 grams benefit the most from surgery within the NICU [3-5]. We include all newborns with VLBW as candidates for bedside surgery at our center. As per a recent survey, 85% of newborns undergoing bedside surgery have a birth weight of <1500 grams [4]. This might be due to the fact that the majority of the newborns treated in the NICU belong to this weight category [9]. Additionally, in concordance with our inclusion criteria, it has been depicted that extremely sick babies (of any birth weight) on HFOV, conventional ventilators with high settings, and multiple inotropes also benefit from bedside surgeries [3,5]. It must also be highlighted that the youngest gestational age and minimum birth weight in our cohort were 27 weeks and 500 grams respectively. These findings are similar to a recent study by Herle et al. [3]. 

Around 60% of the bedside procedures performed at our center were for perforation peritonitis or intestinal obstruction. Similar results have been demonstrated by previously published studies [3-5]. Additional bedside procedures that are commonly reported by various centers include clipping/ligation of the PDA, gastroschisis repair, repair of the CDH, insertion of a peritoneal dialysis catheter, etc. [3,4]. In addition, we have also performed two bedside procedures by minimally invasive approach, i.e. cystoscopy with deroofing of ureterocele and diagnostic laparoscopy for distal ileal atresia. It is believed that this approach avoids a large incision of exploratory laparotomy and subsequent exposure of the entire small bowel to the external milieu. This ultimately reduces the chances of hypothermia and fluid-electrolyte imbalance in these newborns.

On analyzing various factors associated with early postoperative mortality, it was found that the only variable having a significant association with unfavorable survival outcomes was the requirement of multiple inotropes and/or HFOV in the perioperative period. To our surprise, neither gestational age nor birth weight had any significant association with early mortality; despite these factors been linked to increased susceptibility of newborns to nosocomial infection. The above can be explained by the low incidence of sepsis in our cohort due to separate nursing of these newborns in the controlled environment of isolation rooms. In contrast, the NICUs where surgeries are performed on the same bed have a higher infection rate and mortality rate [2,10]. 

There are few limitations of this study. Firstly, this is a retrospective study with a small sample size. Secondly, the present study doesn’t compare the characteristics and outcomes of the included newborns with those operated for similar conditions within the OR [2,3]. As depicted by previously published studies, a comparison of both the groups would have provided additional insight into the safety and feasibility of the bedside surgery approach. However, we believe, the comparison of both the groups should not be performed due to the problems of selection and confounding bias. The clinical characteristics of the newborns within the bedside surgery group (like preterm, very low birth weight, critically ill, etc.) are different from those of the OR group, leading to poorer survival outcomes in the former. Finally, unlike other studies, we have not adopted a scoring system for the meticulous selection of the cases for bedside surgery [11,12].

Despite the above limitations, we report favorable one-month and overall survival of the newborns operated within the NICU. Although we have not compared the perioperative physiological characteristics of our cohort with the already published literature, we believe this favorable outcome is mainly a result of the ideal NICU arrangement, i.e. the newborn is operated in a separate, sterile, isolation room that is fully equipped [8]. It not only reduces the incidence of postoperative infection in highly-susceptible preterm newborns but also causes minimal disruption of the routine NICU services. Another factor contributing to the low mortality rate in our study may be the inclusion of VLBW and not ELBW (extremely low birth weight) babies, as the latter have poorer survival outcomes.

## Conclusions

Performing surgeries within the NICU is a safe and feasible alternative to OR in at-risk newborns. One-month survival of as high as 84% can be achieved if these surgeries are performed in an ideal setting, i.e. sterile isolation rooms. The only factor significantly associated with early mortality in our limited cohort of newborns was the requirement of multiple inotropes and/or HFOV.
